# Depletion-Induced Chiral Chain Formation of Magnetic Spheres

**DOI:** 10.3390/ma14030507

**Published:** 2021-01-21

**Authors:** Sandrine M. F. Heijnen, Patrick van Vliet, Bonny W. M. Kuipers, Albert P. Philipse, Andrei V. Petukhov, Samia Ouhajji

**Affiliations:** Van ’t Hoff Laboratory for Physical and Colloid Chemistry, Debye Institute for Nanomaterials Science, Utrecht University, Padualaan 8, 3584 CH Utrecht, The Netherlands; Sandrine.Heijnen.19@ucl.ac.uk (S.M.F.H.); P.vanVliet@students.uu.nl (P.v.V.); B.W.M.Kuipers@uu.nl (B.W.M.K.); A.P.Philipse@uu.nl (A.P.P.)

**Keywords:** chirality, depletion-interaction, superparamagnetic colloids

## Abstract

Experimental evidence is presented for the spontaneous formation of chiral configurations in bulk dispersions of magnetized colloids that interact by a combination of anisotropic dipolar interactions and isotropic depletion attractions. The colloids are superparamagnetic silica spheres, magnetized and aligned by a carefully tuned uniform external magnetic field; isotropic attractions are induced by using poly(ethylene oxide) polymers as depleting agents. At specific polymer concentrations, sphere chains wind around each other to form helical structures–of the type that previously have only been observed in simulations on small sets of unconfined dipolar spheres with additional isotropic interactions.

## 1. Introduction

Chirality is a property of geometrical objects, the mirror image of which is not superimposable with the original [1]. Kelvin coined the term ‘chirality’, from the Greek $\chi \epsilon \overset{´}{\iota }\rho$ for hand–perhaps the most familiar example of a chiral object [2,3]. While physical properties, such as melting temperature or colour, of left- and right-handed molecules are identical, the handedness of a molecule sensitively affects its odour, toxicity and optical activity for instance [1,2]. Chiral isomers of the molecule limonene, for example, are responsible for the distinct aroma of lemons versus that of oranges [4].

The absence of inversion symmetry manifests itself over many length scales; from the double helix of DNA to the coiling of cucumber tendrils [5] and chiral liquid crystals [6,7,8]. Whereas achiral rods can form a nematic phase with long-range orientational order but no positional ordering, chiral rods can transform the nematic into a chiral nematic (also known as cholesteric) phase in which the orientation of the rods rotates over a macroscopic distance.

Two systems exhibiting spontaneous chiral symmetry breaking were studied theoretically by Pickett et al. [9]. One system employs close-packed isotropic spheres that form helical structures upon cylindrical confinement due to geometrical restrictions [10,11]. Macroscopically this can be demonstrated by filling a measuring cylinder with marbles of the proper size ratio. Experimentally this was realized by the formation of helical chains from colloidal spheres confined in V-grooves by capillary forces [12]. Jiang et al. demonstrated this helical chain formation via the co-assembly of colloids and microtubes [13] and Ouhajji et al. developed a method to fix the assembled chiral colloids together [14,15].

The second system comprises unconfined dipolar hard spheres with an additional isotropic attraction that were found to exhibit chiral ground states in simulations [9]. These simulations were performed for chains containing 15 aligned spheres with embedded dipole moments which, for certain values of the strength and range of the attraction, formed helical structures. The observed chiral structures mainly consisted of three chains winding around each other.

Here, we demonstrate experimentally the arrangement of unconfined dipolar particles into helical chains due to isotropic attraction caused by depleting polymers. To this end, we employ negatively charged superparamagnetic silica spheres as dipolar spheres. Salt is added to reduce the Debye length and to effectively obtain dipolar hard spheres. Addition of the polymer poly(ethylene oxide) generates an additional attraction between the particles in the form of depletion interaction. An external homogeneous magnetic field is applied to align the magnetic particles in a sea of depletants with varying concentrations.

## 2. Materials and Methods

### 2.1. Materials

Superparamagnetic silica colloids in water with a diameter (specified by the manufacturer) of 0.51 μm ± 0.03 μm and iron oxide content ≥30% were purchased from microParticles GmbH (Berlin, Germany). Poly(ethylene oxide) (PEO, 600 kDa) was purchased from Sigma-Aldrich (St. Louis, MO, USA), sodium chloride (p.a.) from Merck (Kenilworth, NJ, USA) and sodium azide (≥99%) from Fisher Scientific (Hampton, NH, USA). All chemicals were used as received. All water used was purified by a Milli-Q water purification system.

### 2.2. Magnetic Field

A Helmholtz cube was developed for in situ observation of colloids in a homogeneous magnetic field, see Supporting Information and Figure S1. Three pairs of Helmholtz coils were arranged orthogonally producing a three-dimensional magnetic field.

### 2.3. Optical Microscopy

Optical microscopy images were obtained using a Nikon Eclipse Ti-E inverted microscope equipped with a DMK 23UX174 Digital Camera (The Imaging Source Europe GmbH, Bremen, Germany). A Nikon CFI Apo TIRF objective (100× magnification, N.A. 1.49) was used. Pictures were recorded in bright field mode. Samples were prepared by mixing desired amounts of stock solutions of superparamagnetic silica spheres, PEO and salt in vials and hollow glass tubes (0.1 × 2 × 50 mm, VitroCom) were filled with these mixtures by capillary action. The tubes were sealed with optical adhesive (Norland 81) that was cured with UV-light (wavelength of 365 nm, 6 W UVP UVGL-58 lamp) and sealed with a layer of nail polish to prevent solvent evaporation. Samples consisted of varying concentrations superparamagnetic colloids and PEO, and 10 mm sodium chloride. Sodium chloride was occasionally (partially) replaced by sodium azide (while keeping the total ionic strength constant) to suppress bacterial growth.

## 3. Results and Discussion

### 3.1. Model System

We used negatively charged superparamagnetic silica spheres as dipolar spheres to determine experimentally if unconfined dipolar particles with an isotropic attraction arrange into helical chains. Two batches of commercial superparamagnetic silica spheres were used with diameters (2$R_{c}$) of 566 ± 18 nm and 548 ± 14 nm, as determined with TEM (see Figure S4a in Supporting information). In the absence of an external magnetic field, well-dispersed spheres can be observed in water, see Figure S4b. 10 mM salt was added to reduce the Debye screening length to 3 nm (see Appendix A). At this ionic strength, colloidal stability was maintained while the double-layer interactions were considerably screened, resulting in effectively dipolar hard spheres.

**Figure 1:**
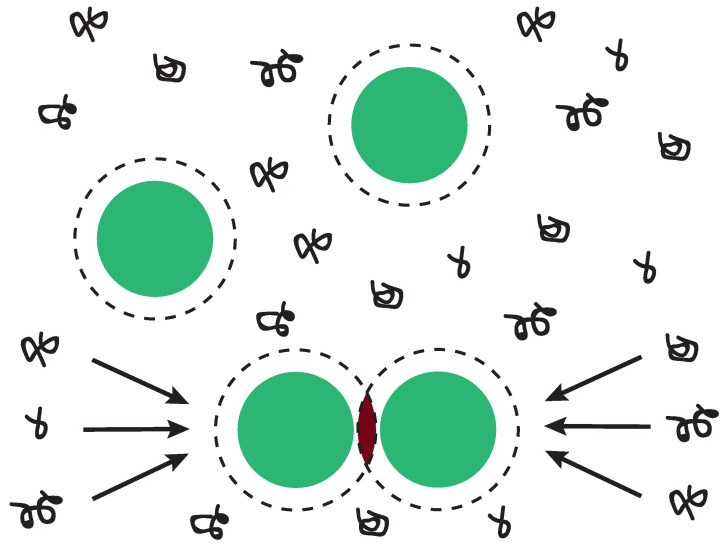
Schematic depiction of depletion interaction. In a mixture of colloidal spheres and non-adsorbing polymer, a depletion layer arises around the spheres as indicated by the dashed circles [16]. The centre of mass of the polymer is excluded (depleted) from this layer due to loss of configurational entropy. When two or more depletion layers overlap, the free volume available for the polymers increases. The polymers thus exert a net osmotic pressure on the colloids forcing the particles together.

In the numerical simulations by Pickett et al. [9], the applied pairwise attractive potential was modelled by

(1) $$U_{\mathrm{attr}}\left(r\right)=\left\{\begin{array}{cc} -\epsilon \frac{R-r}{R-2R_{c}} & 2R_{c}\leq r\leq R\\ 0 & \mathrm{otherwise} \end{array}\right.,$$

as a function of the distance *r* between two spheres. Here, ϵ defines the strength and *R* the range of the attraction. Experimentally, isotropic attraction between particles can be achieved through the addition of non-adsorbing polymers. In a mixture of colloids and non-adsorbing polymer, negative adsorption of a polymer chain results in a loss of configurational entropy due to a depletion layer around the colloid which the centre of mass of the polymer cannot penetrate, see Figure 1 [16]. To minimize their free energy, the polymers exert a net osmotic pressure on the colloids forcing the particles together. This apparent attraction between the colloidal particles is named the depletion interaction. It is an apparent attraction as depletion is entropically driven and arises as a result of purely repulsive colloid-colloid and colloid-polymer interactions. The range of the depletion potential is determined by the size of the depletant, whereas the attraction strength depends on the osmotic pressure and thus on the depletant concentration. Therefore, the strength and the range of the attraction between colloids can be modified independently using depletion forces.

**Figure 2:**
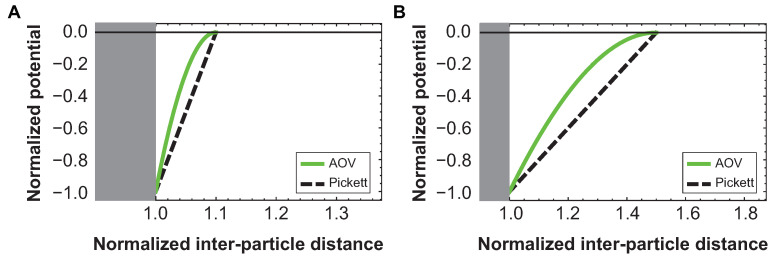
Comparison of potentials. AOV depletion potential and the potential as given in Equation (1) (denoted here as Pickett potential) normalized to the contact potential as a function of normalized inter-particle distance for (**A**) q = 0.1 and (**B**) q = 0.5. For small q, the two potentials are fairly similar.

In Figure 2 it is shown that in the limit of small *q*, the Asakura-Oosawa-Vrij (AOV) depletion potential [16,17] approximates the potential given in Equation (1) reasonably well. To compare the two potentials, the following conversion was applied: R=q+1. As depletant, PEO was used with a molecular weight of 600 kDa and a (calculated) radius of gyration $R_{g}$ of 50 nm (see Appendix A) [18]. The size ratio *q* of the polymer-colloid mixture is thus equal to $R_{g}/R_{c}=0.18$.

An external magnetic field is applied to align the magnetic particles in a sea of depletants with varying concentrations. A Helmholtz cube was developed for in situ observation of colloids in a homogeneous magnetic field, see Supporting Information and Figure S1, as an inhomogeneous magnetic field resulted in (reversible) lateral aggregation (see Supporting Information and Figures S2 and S3).

### 3.2. Observations

**Figure 3:**
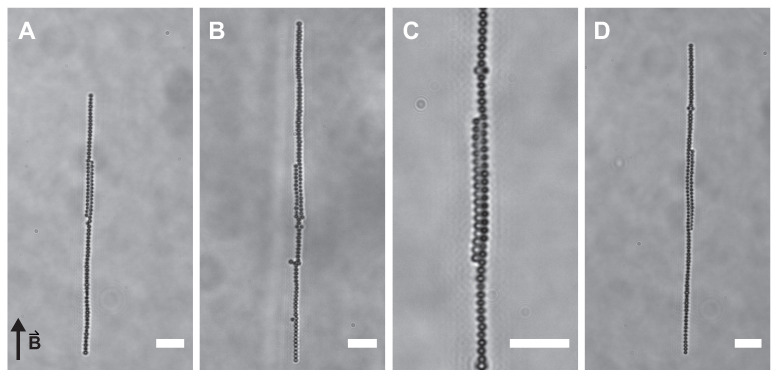
Linear sphere chains. In a homogeneous magnetic field of 3 mT, linear sphere chains are formed for all studied concentrations of PEO. (**A**) 0 mg/mL, (**B**) 0.10 mg/mL, (**C**) 0.14 mg/mL and (**D**) 0.16 mg/mL PEO. Scale bars are 5 μm. All images were taken approximately one hour after switching the magnetic field on. The arrow indicates the direction of the applied field in all images.

**Figure 4:**
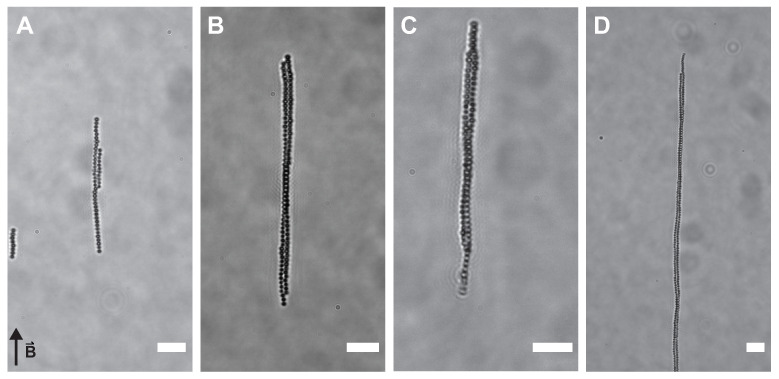
Helical sphere chains. In a homogeneous magnetic field of 1.4 mT, long chains of spheres wind around each other forming helical chains in the presence of depletant. (**A**) 0 mg/mL, (**B**) 0.10 mg/mL, (**C**) 0.14 mg/mL and (**D**) 0.16 mg/mL PEO. Scale bars are 5 μm. All images were taken approximately one hour after switching the magnetic field on. The arrow indicates the direction of the applied field in all images.

Samples containing 50 mg/mL superparamagnetic silica particles, 10 mM salt and PEO concentrations ranging from 0 mg/mL to 1 mg/mL were placed in the homogeneous magnetic field of the Helmholtz cube. This polymer concentration corresponds to depletion potentials of 0.5 to 6 k_B_T (see Appendix A). A magnetic field of 3 mT results in an interaction strength on the order of −65 k_B_T (assuming point dipole-dipole interactions), which is sufficient to induce chain formation of the superparamagnetic silica spheres. However, in a homogeneous magnetic field of 3 mT, mixtures of magnetic silica spheres, salt and depletant do not form chiral structures, see Figure 3. By lowering the magnetic field strength to 1.4 mT (dipolar hard sphere interaction at contact of −4 k_B_T), two adjacent linear sphere chains wind around each other at three depletant concentrations, see Figure 4 and Supporting Videos. Typically, images and videos were recorded after one hour of equilibration. Chains start to form immediately upon switching the magnetic field on. After approximately one hour, nearly all particles in dispersion are found in chains (see Supporting Video). Upon removing the magnetic field, structures fall apart immediately in this completely reversible process (see Supporting Video). Both left- and right-handed structures occur with equal probability as expected for a spontaneous symmetry breaking process. Most structures showed one twist with the pitch increasing with the length of the chains and thus the number of spheres per chain.

These long chains of entwined spherical particles did not form in the absence of depletant or at higher magnetic field strengths. In a magnetic field of 3 mT, the dipole-dipole interactions are much stronger compared to the depletion potential and rigid linear chains dominate. More dynamic structures can be obtained in a weaker magnetic field, due to competing dipolar and depletion interactions, allowing the exploration of more configurations and ultimately the formation of helical sphere chains. In a helical arrangement, the particles accommodate more neighbours leading to an increase in the entropy of the depleting agent while winding allows the system to minimize the magnetic interaction energy.

### 3.3. Comparison with Simulations

Upon introduction of an additional isotropic attraction, unconfined dipolar spheres arrange into helical bundles, both in silico and in vitro. However, whereas in the simulations three chains of spheres were found to wind around each other, in the experiments no more than two chains of spheres arranged to form a twisted structure. Even at higher magnetic field strengths, not more than two parallel chains were observed. Thereby, the simulations were limited to 15 particles in total; a substantially larger amount of particles were employed experimentally leading to vastly larger helical structures.

**Figure 5:**
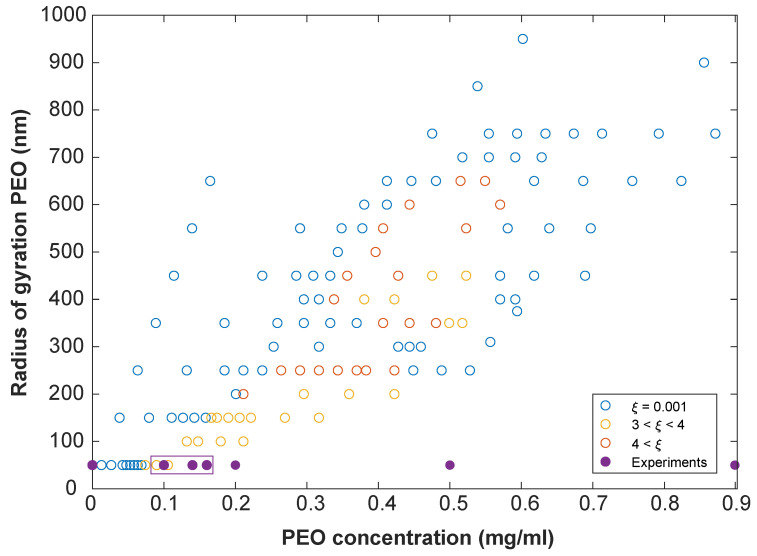
Phase diagram. Spontaneous symmetry breaking as a function of the radius of gyration and the concentration of PEO. Open circles indicate the theoretical results obtained by Pickett et al. [9]. These are colour-coded with the chiral order parameter, i.e., the amount of chirality of a structure. The blue open circles are achiral states whereas the yellow and orange open circles indicate two different chiral states. The filled circles are the experimental data points as discussed in this Article. The points enclosed inside the box are chiral, whereas those outside of the box are achiral. One point overlaps. Experimental data points were obtained in a homogeneous magnetic field of 1.4 mT with silica particle concentration of 50 mg/mL and 10 mM salt.

In Figure 5, our experimental results are presented in a phase diagram together with the simulation results of Pickett et al. [9]. Only a small part of the available phase space could be probed by the chosen experimental model system as it is limited to small q. Employing larger PEO polymers would result in a deviation from the required pairwise potential as discussed previously. Likewise, smaller dipolar spheres would impede characterization with optical microscopy. The experimental helical structures are found close to the theoretically predicted conditions with one point overlapping in the phase diagram. This indicates that a correct model system was chosen. Small deviations can be explained by the discrepancy in the applied potentials.

## 4. Conclusions

We have shown experimentally the spontaneous formation of chiral chains from dispersions of magnetic colloids and depleting polymers in an external magnetic field. For specific depletant concentrations, chains of spheres are found to wind around each other forming helical structures. These structures maximize overlap of exclusion zones between neighbouring spheres and, hence, lower the free energy of the system. Our findings confirm for the first time the theoretical prediction that unconfined dipolar spheres with an additional isotropic attraction form helical structures. A topic for future investigations is the functionalization of the superparamagnetic silica spheres with photo-responsive molecules to obtain permanent sphere chains. Upscaling of the permanently fixed chiral chains could then lead to the study of colloidal chiral liquid crystals.

## Data Availability

The data presented in this study are available on request from the corresponding author.

## Supplementary Materials

The following are available online at https://www.mdpi.com/1996-1944/14/3/507/s1, Figure S1: Helmholtz cube, Figure S2: linear sphere chains, Figure S3: helical sphere chains, Figure S4: superparamagnetic silica spheres, Table S1: properties of the three pairs of Helmholtz coils, Video S1: formation of linear chains in a magnetic field of 3 mT (video is sped up, total duration: 1 h), Video S2: chiral chain of magnetic spheres in magnetic field of 1.4 mT, Video S3: long chiral chain of magnetic spheres in magnetic field of 1.4 mT, Video S4: chiral chain falling apart upon turning off the magnetic field.

## Appendix A. Calculations

### Appendix A.1. Debye Screening Length

The electric potential profile surrounding a colloidal particle approaches zero at a distance of several times the Debye screening length $\kappa^{-1}$ given by

(A1) $$\kappa^{-1}=\sqrt{\frac{\epsilon_{0}\epsilon_{r}k_{B}T}{2e^{2}n_{0}}}.$$

Here, $\epsilon_{0}$ is the vacuum permittivity, $\epsilon_{r}$ is the dielectric constant of the medium, $k_{B}$ is the Boltzmann constant, *T* is the absolute temperature, *e* is the elementary charge and $n_{0}$ the particle density at the point where the field is zero. In water at 25 °C, Equation (A1) reduces to

(A2) $$\kappa^{-1}=\frac{0.30}{\sqrt{c}}\;\mathrm{nm},$$

with *c* the salt concentration in mol/L [19].

### Appendix A.2. Radius of Gyration

Devanand and Selser studied the behaviour of PEO in water with static and dynamic light scattering measurements [18]. In a good solvent, the following relation holds between the radius of gyration $R_{g}$ and molecular weight $M_{w}$ (in g/mol) of PEO.

(A3) $$R_{g}=0.215M_{w}^{0.583\pm 0.031}\;Å$$

### Appendix A.3. Depletion Potential

The depletion potential between two spheres W(h) due to ideal polymers is, in the Derjaguin approximation, given by [16]

(A4) $$W\left(h\right)=-n_{b}k_{B}TR_{c}R_{g}^{2}\left(4\pi \ln2-4\sqrt{\pi }\frac{h}{R_{g}}+\frac{\pi }{2}\frac{h^{2}}{R_{g}^{2}}\right).$$

Here, $n_{b}$ is the bulk number density of ideal polymers, $R_{c}$ is the radius of the spheres, $R_{g}$ is the radius of gyration of the polymer and *h* is the separation between two spheres. The strength of the interaction or the depth of the potential ϵ at contact (h=0) is thus

(A5) $$\epsilon =\frac{W(h)}{k_{B}T}=-n_{b}R_{c}R_{g}^{2}4\pi \ln2.$$
