# Supplementary material for: Depletion-Induced Chiral Chain Formation of Magnetic Spheres
Source: Materials (Basel). 2021 Jan 21;14(3):507. doi: 10.3390/ma14030507 (PMC7865936; doi:10.3390/ma14030507)
Supplement: Supplementary file 1 [file materials-14-00507-s001.zip › materials-1070195-SM-update/materials-1070195_SI-update.pdf]

Supplementary Materials: Depletion-Induced Chiral Chain Formation of Magnetic Spheres

Sandrine M. F. Heijnen, Patrick van Vliet, Bonny W. M. Kuipers, Albert P. Philipse, Andrei V. Petukhov* and Samia Ouhajji †,*

1. Homogeneous Magnetic Field

A Helmholtz cube was developed for in situ observation of colloids in a homogeneous magnetic field, see Figure S1. Three pairs of Helmholtz coils were arranged orthogonally producing a three-dimensional magnetic field. Two circular coils form the inner Helmholtz pair that provides the magnetic field in the z-direction (vertically). This circular geometry was chosen to minimize the diameter (and consequently maximize the field strength), and to ensure that the cylindrical microscope objective can fit inside the Helmholtz cube. The cylindrical coils are positioned concentrically on the optical axis of the microscope, at a distance $d = R$, with R the radius of the coils. The magnetic field B at this distance, is given by [1]

$$B = \frac{8\mu_0 N}{5\sqrt{5}R} I, \tag{1}$$

where μ_0 is the permeability of free space ($4\pi \cdot 10^{-7}$ Tm/A), N is the number of turns of copper wire and I is the current running over the coils. The gradient of the field $\partial B/\partial z$ and the second derivative of the magnetic field $\partial^2 B/\partial z^2$ both equal zero exactly in the centre of the coils.

The middle and outer Helmholtz pairs are formed by two sets of square coils, a more convenient shape in practice. The distance d between each set of coils is equal to 0.5445 times the length of the square sides [2,3]. As with the circular coils, both the gradient and the second derivative of the magnetic field vanish precisely in between the coils. For the square coils, the magnetic field $\vec{B}$ can be calculated using the Biot-Savart law [1,4]

$$\vec{B}(\vec{r}) = \frac{\mu_0}{4\pi} I \oint \frac{d\vec{l} \times \vec{r'}}{r'^3}. \tag{2}$$

The integration is over the length $\vec{l}$ of the sides of the squares carrying the steady current I and $\vec{r'}$ is the vector between the observation point $\vec{r}$ and the element of the coil $\vec{l}$. The fluctuations of the magnetic field are on the order of 0.5%.

An xy translation stage was designed to place a microscope sample in the field of view of the microscope and in the centre of the Helmholtz cube. The Helmholtz frame and the sample holder were printed by a 3D printer. Copper wires were wound on the coils by the company Wikkelbedrijf Rijswijk B.V. in Driebruggen, the Netherlands. The diameter of the round wires is 0.71 mm, allowing a DC current of maximum 1.55 A in the absence of external cooling. This current generates a quasi-static magnetic field in air of at least 3 mT in the x- and y-directions and 6 mT in the z-direction. The properties of the Helmholtz coils are summarized in Table S1.

Table 1: Properties of the three pairs of Helmholtz coils. The field denotes the maximum field strength in the absence of external cooling.

Coil pair	Field direction	Geometry	Turns	Field (mT)
Outside	X	Square	170	3.2
Middle	Y	Square	140	3.2
Inside	Z	Circular	170	6.2

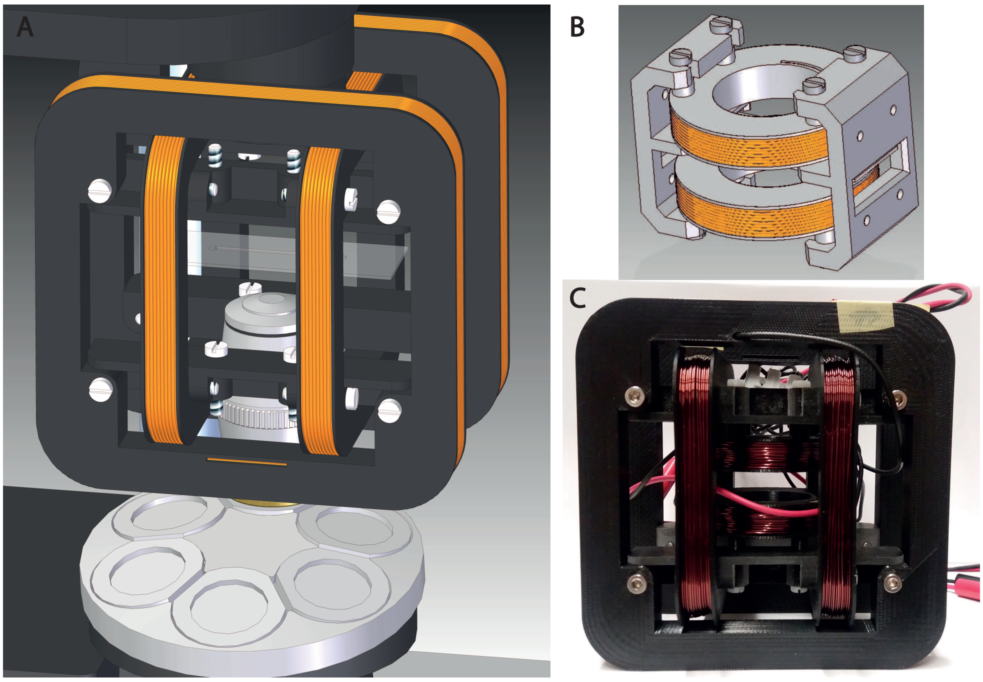

Figure S1. Helmholtz cube. (**A**) Schematic representation of two pairs of square Helmholtz coils encompassing a microscope sample and objective, co-axial to the optical axis of an inverted microscope. The midpoint of the three orthogonal coils is positioned at the focal point of the microscope. (**B**) A schematic of the inner Helmholtz pair formed by two circular coils. (**C**) Photo of the realised Helmholtz cube.

2. Inhomogeneous Magnetic Field

An external horizontal inhomogeneous magnetic field was induced by two permanent magnets (Geomag, Novazzano, Switzerland) placed equidistantly from the sample contained in a capillary. The distance between the magnets was chosen such that the strength of the magnetic field in the sample was about 3 mT as determined with a gaussmeter. Samples were studied containing 50 mg/mL superparamagnetic silica particles, 10 mM sodium chloride and PEO concentrations ranging from 0.1 mg/mL to 1 mg/mL (corresponding depletion potential of 0.5 to 6 k_BT). Mostly linear chains of spheres were observed and occasionally chains formed ribbons with widths larger than one particle diameter by (reversible) lateral aggregation [5], see Figure S2. For two specific PEO concentrations helical structures were observed, see Figure S3. These long chains of entwined spherical particles did not form in the absence of depletant.

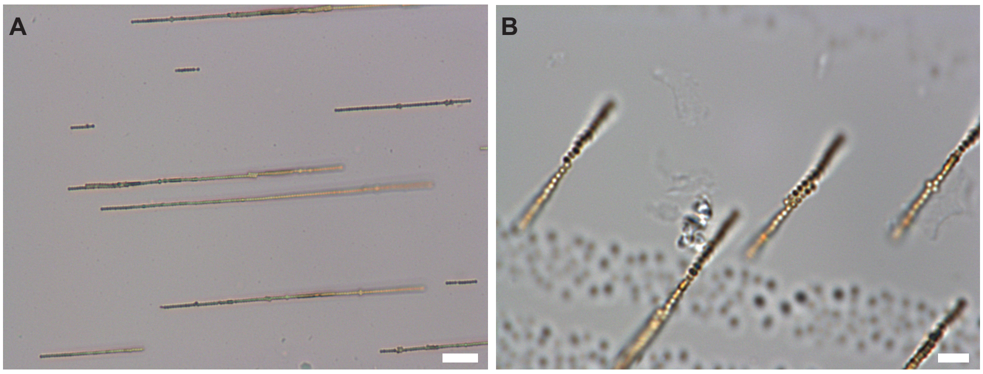

Figure S2. Linear sphere chains. Linear chains of magnetic spheres were formed for most concentrations of PEO. Lateral aggregation of different chains was observed occasionally. (**A**) 0.13 mg/mL PEO; scale bar is 5 μm. (**B**) 1 mg/mL PEO; scale bar is 3 μm.

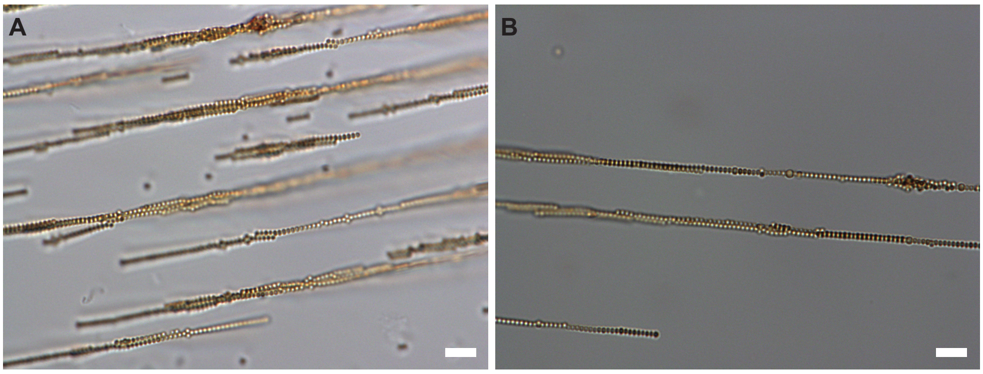

Figure S3. Helical sphere chains. At two concentrations of PEO, (**A**) 0.14 mg/mL and (**B**) 0.16 mg/mL, linear chains of spheres were found to wind around each other. Scale bars are 3 μm.

3. Particles

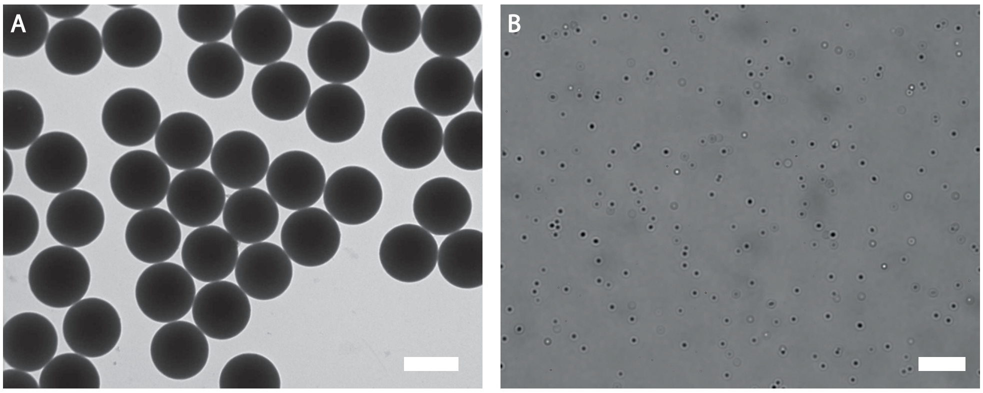

Figure S4. Superparamagnetic silica spheres. Representative (**A**) TEM and (**B**) optical microscopy images in the absence of an external magnetic field. Scale bars are 500 nm and 20 μm respectively.

References

1. Griffiths, D.J. *Introduction to Electrodynamics*; Prentice Hall, 1999.
2. Abbott, J.J. Parametric design of tri-axial nested Helmholtz coils. *Rev. Sci. Instrum.* **2015**, *86*, 054701.
3. Rudd, M.E.; Craig, J.R. Optimum spacing of square and circular coil pairs. *Rev. Sci. Instrum.* **1968**, *39*, 1372–1374.
4. Jackson, J.D. *Classical Electrodynamics*; Wiley, 1999.
5. Darras, A.; Fiscina, J.; Pakpour, M.; Vandewalle, N.; Lumay, G. Ribbons of superparamagnetic colloids in magnetic field. *Eur. Phys. J. E* **2016**, *39*, 47. doi:10.1140/epje/i2016-16047-0.
